# Identification of Korean cancer survivors’ unmet needs and desired psychosocial assistance: A focus group study

**DOI:** 10.1371/journal.pone.0228054

**Published:** 2020-01-16

**Authors:** Joo Young Kim, Mison Chun, Sang-Won Kim, Joonsup Song

**Affiliations:** 1 Department of Psychology, Duksung Women’s University College of Social Science, Seoul, Republic of Korea; 2 Department of Radiation Oncology, Ajou University School of Medicine, Suwon, Republic of Korea; 3 Department of Radiation Oncology, Konyang University College of Medicine, Daejeon, Republic of Korea; 4 Department of Pediatrics, Konkuk University Chungju Hospital, Chungju, Republic of Korea; University of Auckland, NEW ZEALAND

## Abstract

**Purpose:**

This qualitative study identifies difficulties and unmet needs in psychosocial aspect that Korean cancer survivors reported.

**Materials and Methods:**

We enrolled 18 cancer survivors who agreed to participate in the focus groups. Each focus group consisted of four to six cancer survivors, considering homogeneity of sex and age. Participants were asked to freely describe the practical difficulties they faced and their unmet needs when living as cancer survivors. A cross-case interview analysis was used to identify major themes. Consensual qualitative research analysis was applied to complement the objectivity of results obtained from participants’ interviews.

**Results:**

We identified three major themes: 1) shifts what cancer connotes, 2) development of government policies regarding integrative management for cancer survivors, and 3) preparing for cancer survivors’ future through vocational rehabilitation or career development.

**Conclusion:**

Korean cancer survivors had difficulties in psychosocial adjustment even after the completion of anti-cancer treatments. We identified several unmet needs among participants who were living as cancer survivors. This qualitative study may expand the view of cancer survivorship in Korea by incorporating their voices and experiences to facilitate the development of a more holistic cancer survivorship program.

## Introduction

The number of cancer survivors is significantly increasing owing to early diagnosis and advances in treatment techniques in Korea. In 2011–2015, the 5-year relative survival rate for all cancers in Korea was 70.7%, there were approximately 1.6 million cancer survivors as of 2015 [1]. This increment has led health care planners and policymakers to facilitate integrative care for cancer survivors. Cancer survivors experience decreased health-related quality of life (HR-QOL) after the completion of aggressive anti-cancer treatments, including limited physical activity and late sequelae due to anti-cancer treatment as well as loss of self-esteem, depression, fear of recurrence, financial burden, negatively affected work performance and relationship difficulties [2, 3]. As the number of long-term cancer survivors increases, HR-QOL emerges as one of major concerns along with survival. Cancer patients were found to have relatively lower HR-QOL than other patients with chronic illnesses [4].

However, existing survivorship care is primarily weighted toward follow-up examination and prevention of recurrence and has shown little interest in HR-QOL, particularly with respect to psychosocial adjustment. Therefore, novel cancer survivorship plans with continuous physical and psychosocial health care are needed for improving HR-QOL of cancer survivors [5].

Better HR-QOL can be achieved if cancer survivors learn how to navigate psychosocial adjustment which is the ability of self-control to cope with various stress from altered physical appearance and surrounding circumstances after anti-cancer treatments. To support psychosocial adjustment of cancer survivors, it is important to resolve their unmet needs.

There have been several quantitative studies on the psychosocial difficulties and unmet needs of Korean cancer survivors [6, 7]. However, quantitative studies have limitations in identifying unmet needs, as respondents’ voices and experiences are not covered intimately. This qualitative study aimed to explore the difficulties in daily lives experienced by Korean cancer survivors and to identify their unmet needs through direct contact with them.

## Material and methods

An Institutional Review Board of Ajou University School of Medicine approved this study before participants’ enrollment. Participants were recruited from outpatient clinics and integrative care centers (Gyeonggi Regional Cancer Center) where educational and supportive programs were held for cancer patients, regardless of their place of treatment.

### Inclusion criteria

Cancer survivors were eligible for enrollment in the study if 1) they had completed treatment with a curative aim, 2) they had no evidence of disease at the time of recruitment, 3) they were able to communicate in Korean, and 4) they were able to comprehend the informed consent. Cancer survivors who were on hormonal or targeted therapy were also permitted to participate. There were no restrictions on inclusion based on the primary sites of cancer. A total of 18 cancer patients agreed with the consent form with full understanding of the interview process and the need for recording. Their characteristics are summarized in Table 1. The median interval from the first diagnosis to participation in the study was 3 years (range, 1–11 years) and 4 participants had survived for at least 5 years.

**Table 1:** Participants’ characteristics.

No.	Sex	Age	marital status	Primary site	Recurrence	Interval from diagnosis (year)	Primary earner	Main stress factor
1	F	56	Y	ovary	once	4	spouse	appearance
2	F	55	Y	breast	N	2	spouse	relationship
3	F	58	Y	cervix	N	2	spouse	health
4	F	55	Y	breast	N	3	dual	spouse
5	F	44	N	breast	N	1	self	recurrence
6	M	64	Y	stomach	N	4	self	spouse
7	M	73	Y	lung	N	4	pension	health
8	M	65	S*	thyroid	N	7	self	health
9	F	38	Y	cervix	once	3	spouse	recurrence
10	F	41	Y	cervix	once	10	spouse	health
11	F	40	N	ovary	once	2	Self	finance
12	F	39	N	breast	N	2	spouse	health
13	F	34	N	brain	twice	5	parent	health
14	F	49	Y	pancreas	N	1	spouse	recurrence
15	M	48	Y	kidney	once	3	self	finance
16	M	55	D	rectum	N	3	self	aging
17	M	36	Y	rectum	N	2	spouse	health
18	M	43	Y	kidney	twice	11	self	finance

S*, separation

D, divorce

### Focus group procedure

Semi-structured questionnaires for the focus groups were developed by the main researcher, based on a previous study. The list of question was then reviewed and modified by an experienced focus groups moderator and an external consultant with specialty in oncology. The questionnaires were constructed as described by Krueger and Casey [8]; 1) opening questions, 2) transition questions, 3) key questions, and 4) ending questions (Table 2).

**Table 2:** List of questionnaires.

Type of questionnaires	Questionnaires
Opening	1. What kinds of medical assistance would you want as a cancer survivor?
Transition	2. What are some general difficulties you face as a cancer survivor?
Main	3. Do you struggle with any physical difficulties? If so, what are they?
	4. Are you battling any psychological difficulties? If so, what are they? 1) If yes, how are you coping with such problems? 2) Has anyone been providing psychological support? 3) From whom would you want psychological support if help were available?
	5. Has your relationship with your spouse changed after diagnosis? 1) If so, how have you been coping with these changes? 2) What kind of help would you want in this aspect?
	6. Has your relationship with your children changed after cancer diagnosis? 1) If so, how have you been coping with these changes? 2) What kind of help would you want in this aspect?
	7. Have your relationships with your friends, neighbors or religious community changed after cancer diagnosis? 1) If so, how have you been coping with these changes? 2) What kind of help would you want in this aspect?
	8. Have you ever wondered or been concerned with how you ended up with cancer?
	9. Who is responsible for your medical expenses?
	10. What are your expectations regarding upcoming medical expenses (including associated costs)?
Ending	11. Is there anything you would like to add?

Before beginning the focus groups, each participant signed an informed consent and was requested to complete the socio-demographic survey (details of age, gender, marital status, primary household earner, and main stress factor). Given the homogeneity of age and sex, each focus group consisted of 4 to 6 participants in order to ensure that conversation could proceed in a free atmosphere. The interviews were conducted four times at the Gyounggi Regional Cancer Center between March and August 2017, and each interview took approximately 90 minutes. Each focus group session was led by a skillful moderator. The moderator provided detailed clues suitable for the purpose of the study and modified the sequence of questions for the discourse plan. After the interviews were completed, the facilitator summarized the major discussion points to check for additional items or omissions. An assistant (a doctoral student in counseling psychology) also attended the focus groups to observe the group and record non-verbal responses.

To foster an effective discussion focused on the study purpose, a psychologist suggested cues. At the end, the psychologist summarized the issues that were discussed and asked participants to add any remarks or questions. The recording was audiotaped and transcribed verbatim immediately after the completion of each interview by a professional transcriber.

### Analysis process

We conducted this study following 4-step guidance for a systematic analysis process presented by Krueger [9]. In this study, we used cross-case interview to analyze the contents of the focus groups. Consensual qualitative research was undertaken to maintain objectivity [10]. Consensual qualitative research is conducted in 3 general steps: 1) Responses to open-ended questions from questionnaires or interviews for each individual case are divided into domains (or topic areas); 2) core ideas (or abstracts or brief summaries) are constructed for all the material within each domain for each case; and 3) cross-analysis, which involves developing categories to describe consistencies in the core ideas within domains across cases. The consensual analysis team consisted of 3 members (one main researcher, one postdoctoral graduate for psychology, and one PhD in counseling psychology). There was one external auditor who held a PhD in counseling psychology and had a wealth of experience in qualitative research. Each member of the consensual qualitative analysis team independently read the full transcriptions and code. When the meanings were not correct, we checked the field notes and recordings to identify the exact contents. The consensual qualitative analysis team was supervised by the external auditor to check if they had missed any important data, whether there was any bias in the interpretations, where the original data were categorized correctly, and whether the theme was concise and reflected the original data.

## Results

Qualitative analysis identified 3 major themes. The results are summarized in Table 3.

**Table 3:** Results.

Sub-category	Category	Theme
Cancer is a traumatic experience.	Cancer survivors’ view of cancer	Shifts in what cancer connotes
Cancer is a chronic disease.		
Physicians should adjust their view of cancer and suffering patients.	Physicians’ view of cancer	
Physicians should adjust the way in which they treat patients (tone, approach, etc.).		
Physicians should share cancer-related information and knowledge more proactively		
The general public thinks that a cancer diagnosis is a death sentence.	Public awareness of cancer	
It is imperative to run public awareness campaigns for cancer survivors.		
Media (i.e., TV shows & movies) should include more storylines where cancer is treated as a manageable chronic disease.		
There should be a system to provide customized cancer care.	Government policies mandating cancer survivors’ participation in integrative programs	Government policies about integrative management for cancer survivors
Specific medical/psychological programs for each stage of cancer survivorship should be provided.		
Everyone involved in cancer care such as physicians, nurses, and psychologists should team up to take a multidisciplinary approach.		
Cancer care / support services should become more locally available.		
Patients could receive certain additional benefits after opting to educate themselves on their cancer and its treatment.		
Would like to receive rehab-oriented psychological support	Expert-led psychosocial program	
Would like to enroll in family (partner/children) program		
Would like to attend psychology expert-led self-help meetings		
Disability rating to be conditioned on the degree of physical discomfort and types of cancer	Welfare policies tailored to cancer survivors	
Public caregiver services after surgical operations		
Education and mentoring service for children of cancer survivors		
Government policy of childbirth support for young cancer survivors		
Strengthening one’s identity by setting life priorities	Needs for career development	Preparing for cancer survivors’ futures
Relieving pressure as the head of the household		
Preventing depression and inspiring a sense of accomplishment		
Career exploration based on personal capacities of cancer survivors	Planning customized career options including reinstatement relative to stages of cancer treatment	
Development of leisure activities and interests tailored to the survival stages		
Providing information about what survivors can accomplish in terms of career		

### Theme 1: Shifts in what cancer connotes

#### Category 1. Improving cancer survivors’ perceptions of cancer

Participants confessed that they had been obsessed with a wrong perception of cancer before they finished primary anti-cancer treatments. Participants said that the most important thing in returning to their daily lives as cancer survivors was to change their perception about cancer first.

“I think that cancer is similar to being injured in a minor traffic accident” (Participant #4)

“Although it is not over after surgery, I think that cancer is not different from any other disease. I’m managing well while caring for my illness like any other chronic disease.” (Participant #14)

#### Category 2. Changing the perceptions and attitudes of physicians towards cancer survivors

Participants stated that physicians needed to change their view of cancer patients from cure to care. Participants commonly said that during their active treatment course, consultation hours were insufficient and that they had only one-sided conversations with their physicians. Their oncologists authoritatively instructed the treatment modalities that the cancer survivors were going to receive without a full explanation of the rationale and possible side effects.

“The doctor just reviewed the medical chart and talked about my conditions briefly. In addition, he was indifferent to my complaints.” (Participant #8)

“I hope that medical staffs have a better understanding of cancer survivors, and comfort us more wholeheartedly.” (Participant #11)

#### Category 3. Changes in public awareness of cancer

Participants were not free from the social stigma about cancer that their family, neighbors, or friends continued to hold, as indicated in their perception of cancer as a death penalty or an infectious disease. Participants empathized that these prejudice about cancer were the main barriers to psychosocial adjustment. Participants suggested ensuing a public service announcement that cancer survivors were not near death, did not have a contagious disease, and were capable of performing their daily activities.

“My mental stress was greater than the physical symptoms. Occasionally, I was under the illusion that I didn’t have cancer because I did not feel any critical symptoms. But my family and neighbors recognized cancer as an unconditionally fatal disease.” (Participant #17)

“A public advertisement is needed to change the wrong perception that cancer means dying immediately, and the ad should have contents clarifying that cancer is a chronic disease that can be treated.” (Participant #1)

### Theme 2: Government policies about integrative management for cancer survivors

#### Category 1. Government policies mandating cancer survivors’ participation in integrative programs

The participants strongly requested for several government policies for the integrative management of cancer survivors that would ensure medical and psychological services even after cancer survivors were declared as cured of cancer. The policies they suggested included 1) building a system that provides customized care programs reflecting the needs of cancer survivors, 2) development of medical and psychological program according to the survival stage, 3) a multidisciplinary approach consisting of physicians, nurses, and psychologists for holistic care, 4) revitalization of the community cancer center near their residence, and 5) entitlement to national registration as cancer patients for benefits from Korean National Health Insurance, if the patients and their families are obligated to participate in basic education to inform the characteristics of cancer and patients.

“It is necessary for the government or hospitals to check the needs of cancer patients and give them what they require. If I must do what I need by myself, it is too hard. Furthermore, practitioners do not cooperate and annoy very much. It is good to create a total system in one-stop mode to resolve the needs of cancer patients” (Participant #16)

#### Category 2. Psychosocial supportive programs led by experts

Participants experienced anxiety and fear about recurrence or death from initial diagnosis. They felt the lack of programs to support them psychosocially. They stated that a psychological rehabilitation program should be introduced at initial diagnosis to provide cancer survivors with mental stability that ensures more self-confidence. They gave an example of organizing a self-help group program among cancer survivors managed by specialized psychologists.

#### Category 3. Welfare policies tailored to cancer survivors

Participants were dissatisfied with the current welfare policies that concentrate on cancer patients under treatment and felt a lack of welfare benefits after the completion of active treatments. Therefore, they wanted the government to formulate welfare policies tailored to cancer survivors. The representative welfare policies that participants wished for were as follows.

“I would like for cancer survivors to be assigned disability ratings depending on the type of cancer and to be given extra benefits according to the physical discomforts they experience.” (Participant #16)

“I want to be assigned a public caregiver to help with the housework after chemotherapy.” (Participant #12)

“Private lessons and mentoring services are needed for children of cancer survivors who cannot afford educational expenditures.” (Participant #12)

“I wish for a government policy of childbirth support for young cancer survivors.” (Participant #17)

### Theme 3: Preparing for cancer survivors’ future

#### Category 1. Career development and exploration

Participants who were primarily responsible for their household revealed that they struggled with economic burden due to difficulties in maintaining their full-time job during treatment. Other participants not primarily responsible for household wanted to reinforce their self-identity through vocational activities. Therefore, career development and exploration were important issues for cancer survivors to prepare for their future. Participants reported that career development and exploration programs could be helpful for cancer survivors by building their presence, reducing their pressure as head of household, preventing depressive mood disorders, and inspiring a sense of accomplishment.

Participants reported the following:

“I was responsible for earning a living for treatment periods and struggled with economic burden due to difficulties in maintaining a full-time job during treatment.” (Participant #15)

“I really want to work. If I work, I can forget my disease. Also, work can prevent depression. Eventually, I can feel joy, worth, and a sense of accomplishment.” (Participant #13)

#### Category 2. Planning customized career options including reinstatement relative to stages of cancer treatment

Several participants thought that they needed information on re-employment and career exploration based on their ability and talent. In addition, they requested the advice on leisure activities like climbing hills or community service before return to work.

“I want to be busy, but I cannot work much because I lack physical energy. To recharge my batteries, I usually climb a hill in the morning. That helps me a lot. After a little while, I plan to do volunteer work” (Participant #11)

## Discussion

This study was conducted to directly explore the difficulties in their daily lives and issues facing Korean cancer survivors so as to offer assistance to them. In addition, through focus groups, we aimed to identify cancer survivors’ unmet needs in depth by collecting patients’ voices and experiences. The results largely revealed three major themes: 1) Shifts in what cancer connotes, 2) establishment of government policies regarding integrative management for cancer survivors, and 3) cancer survivors’ futures.

First, participants emphasized the need for change in the widespread perception of cancer. Nowadays, cancer has begun to be recognized as a chronic disease requiring long-term management and care because of remarkably improved survival, and not as a matter of life or death. However, this study reveals that in Korea, a cancer diagnosis still causes a serious shock that results in the cancer patient being pronounced dead to themselves and the people around them. Several participants were told by their neighbors that cancer might be a contagious disease and were branded as personae non gratae. Negative public awareness of cancer and/or cancer patients in Korea can be found in a previous study [11]. It is assumed that such a social prejudice towards cancer has been formed because: 1) Overall cancer survival had been low in the past; 2) a number of storylines of TV dramas or movies used to describe the direct connection between cancer and death; and 3) people often thought that cancer might be the price that cancer patients pay for their past misdeeds.

Therefore, the widespread prejudice about cancer should be addressed by education, media, and public relations in order to change the perception of cancer by cancer survivors themselves; all medical staff involved in the diagnosis and treatment of cancer; family members of cancer patients; and the general public. Although cancer should be recognized as a chronic disease, cancer survivors are distinguished from other patients with chronic illness because they consistently suffer from physical discomforts and delayed sequelae such as fatigue, pain, and lymphedema for several months or years after active anti-cancer treatments. In addition, many cancer survivors suffer from mental disorders such as anxiety, depression, and fear of recurrence or death. Due to these problems, the overall HR-QOL of cancer survivors is markedly lower than those of patients with other chronic illnesses [12], suggesting that the perceptions of cancer survivors should not be equated with those of other chronically ill patients.

Many participants experienced discomfort upon receiving medical consultations from physicians. They complained of physicians’ authoritative attitudes toward them and indifference about caring late sequelae related to anti-cancer treatments and mental problems. Lack of information about self-care management from clinicians is commonly found in the previous studies as unmet needs of cancer survivors [13, 14]. This implies that there is a discrepancy in cancer care between physicians’ thoughts and cancer survivors’ expectations. Therefore, all medical practitioners involved should pay attention to cancer survivors’ unmet needs with the aim of providing individualized care.

In the United States, the Institute of Medicine and National Research Council emphasized that cancer survivors should be provided with a survivorship care plan, including 1) specific information about the timing and content of recommended follow-up, 2) recommendations regarding preventive practices and how to maintain health and well-being, and 3) availability of psychosocial services in the community [15]. Based on this report, a number of long-term follow-up models for cancer survivors have been applied in clinical practice, including sharing the medical information of cancer survivors between oncologists and primary care providers, plans for follow-up, monitoring of late toxicities, and education for health promotion [15–17]. Medical staff involved in cancer survivorship in the United States are educated about these models in the refresher training courses. In contrast, little education and training for the management of cancer survivors is provided to medical staff in Korea. To help medical staff better understand cancer survivors’ needs and to educate them about the integrative care to support cancer survivors’ needs, it would be effective if this education course and regular curriculum are prepared in the university or refresher training process.

Integrative management programs for cancer survivors should be developed to simultaneously support medical, psychological, and social services according to the three stages of cancer survival: 1) acute stage, 2) extended stage, and 3) permanent stage [18].

Participants reported that after completing active anti-cancer treatments, they realized the importance of information about the inherent characteristics of cancer, treatment-related toxicities, cancer patient-specific diet, and psychological support from the time of initial diagnosis even though they were not concerned about these aspects during active treatment period. Therefore, at the acute stage, education for cancer patients is needed to inform them about the process of anti-cancer treatments, expected outcomes, possible treatment-related side effects, management of side effects, and diet planning. This education is provided by a multidisciplinary team consisting of oncologists, nurse practitioners, and nutritionists. Psychological services should be accompanied to decrease depression or anxiety for both cancer patients and their caregivers, because caregivers also experience them [19]. Therefore, we suggest that in order to implement an integrative management program from the time of diagnosis, a government policy should be established to ensure that national cancer registration allowing patients to benefit from greater health insurance coverage should require both patients’ and care-givers’ participation in integrative education/management programs along with cancer diagnosis as a prerequisite, as was requested by the participants during interviews.

At the extended stage, the character of cancer changes from an acute to a chronic illness. Many cancer survivors experience long-term physical and psychosocial sequelae at this stage. However, according to the participants in this study, supportive care for cancer survivors is insufficient. Medical supportive care at this stage includes smooth consultations with other departments or primary healthcare providers regarding the management of treatment-related sequelae and the provision of various rehabilitation therapies such as physiotherapy, occupational therapy, and movement therapy for physical adjustment. At this stage, a psychological rehabilitation program should aim to restore cancer survivors’ self-esteem and family relationships. When cancer survivors return to their home and society at this stage, the community cancer center near their residence would be the optimal place to offer integrative management programs, even though many cancer survivors are unaware of community cancer centers because of insufficient publicity.

At the permanent stage, long-term survival is expected, along with low possibility of recurrence. Cancer survivors usually face an economic burden due to job loss during anti-cancer treatment courses or employment difficulties. One of the most important barriers to cancer survivors returning to work is negative public attitudes [20]. Study participants requested various welfare policies tailored to their need, including rating the degree of disability, mentoring services for their school-aged children, dispatch of public caregiver for helping housework, and childbirth support services. These requests can be implemented through utilizing existing welfare policies. For example, The Welfare Law for Persons with Disabilities, enacted in 1981 by the Korean government, stipulates the prioritization of the use of government facilities or other public organizations, disability allowances, rehabilitation counseling and measures for admission to institutions, grants for the educational expenses of children, support of helpers for post-natal care, and support through an activity-supporting allowance. Some of these policies are also aimed at cancer survivors, although they have not been widely publicized. Therefore, it is necessary to promote and improve existing welfare policies to better ensure their availability.

Psychosocial adjustment of cancer survivors implies preparations for the future. Cancer survivors, particularly at a socially active age, often suffer from extreme stress and lose the will to live because they cannot maintain a full-time job and fulfill their family responsibilities. Therefore, through a return to work, cancer survivors can recover economic stability, resume their ordinary lives, and regain self-esteem [21, 22]. A previous study reported that approximately half of cancer survivors could not continue vocational activities during or after anti-cancer treatments [23]. Park et al. reported that cancer patients had a 1.56 times higher risk of job loss after diagnosis than the general population [24]. Cancer survivors fear job loss because their functional activities decline owing to sustained fatigue and physical sequelae, leading to growing state expenditures [25]. Yet, there is no agreed notion of return to work for cancer survivors in Korea. Therefore, the notion of return to work for cancer survivors should be defined before government policies are established. Career development and exploration programs should then be developed to help cancer survivors experiencing job loss to return to work. A vocational rehabilitation program tailored to cancer survivors should also be provided.

This study has several limitations. First, because this is an exploratory study, its results cannot be generalized. Second, we recruited participants irrespective of site of primary tumor, survival period, and age. Cancer survivors’ needs might differ by age, gender, and survival period. Therefore, further studies are needed to address these limitations.

The significance of this study is highlighted by the fact that it attempts to explore the Korean cancer survivors’ unmet needs and concern. This study can serve as a basis for creating government policies on the integrative management of cancer survivors and development of an integrative program. Finally, because this study suggests ways for cancer survivors to resume daily activities, further studies can be designed to offer practical assistance to cancer survivors.
